# Tubulointerstitial nephritis and uveitis syndrome in children: report of three cases

**DOI:** 10.1590/2175-8239-JBN-2018-0015

**Published:** 2018-06-18

**Authors:** Cátia Pereira, Joana Gil, Inês Leal, Patrícia Costa-Reis, José Eduardo Esteves Da Silva, Rosário Stone

**Affiliations:** 1 Serviço de Pediatria Médica, Departamento de Pediatria, Hospital de Santa Maria, Centro Académico de Medicina, Universidade de Lisboa, Lisboa, Portugal.; 2 Serviço de Oftalmologia, Hospital de Santa Maria, Centro Académico de Medicina, Universidade de Lisboa, Lisboa, Portugal.; 3 Centro de Estudos das Ciências da Visão, Faculdade de Medicina, Universidade de Lisboa, Lisboa, Portugal.; 4 Unidade de Nefrologia e Transplantação Renal, Serviço de Pediatria Médica, Departamento de Pediatria, Hospital de Santa Maria, Centro Académico de Medicina, Universidade de Lisboa, Lisboa, Portugal.

**Keywords:** beta 2-Microglobulin, Nephritis, Interstitial, Uveitis, Microglobulina-2 beta, Nefrite Intersticial, Uveíte

## Abstract

Tubulointerstitial nephritis and uveitis syndrome is a rare and probably underdiagnosed condition. Renal and ocular manifestations may not occur simultaneously, making the diagnosis more difficult. Nephritis may be asymptomatic; therefore, renal function evaluation is essential for diagnosis. Urinary β2-microglobulin levels may be particularly useful. Uveitis, mostly anterior, nongranulomatous and bilateral, occurs usually after the onset of nephritis. Treatment includes corticosteroids and, eventually, other immunosuppressant agents. Renal disease is usually benign and resolves spontaneously or after treatment with systemic corticosteroids. Uveitis, however, may be chronic or recurrent. The authors described the cases of three pediatric patients diagnosed with tubulointerstitial nephritis and uveitis syndrome. The goal of this paper was to warn the medical community over the need to screen patients with uveitis for renal disease.

## RESUMO

A síndrome nefrite tubulointersticial e uveíte é uma doença rara, provavelmente subdiagnosticada. As manifestações renais e oculares podem não ocorrer simultaneamente, tornando o diagnóstico mais difícil. A nefrite é geralmente assintomática, tornando fundamental a avaliação da função renal em doentes com uveíte. O doseamento da excreção urinária de β2-microglobulina é particularmente útil para o diagnóstico. A uveíte, tipicamente anterior, não granulomatosa e bilateral, manifesta-se após a nefrite na maioria dos casos. O tratamento inclui corticoides e, por vezes, outros imunossupressores. A doença renal tem evolução benigna, resolvendo-se espontaneamente ou com terapêutica com corticoides sistêmicos na maioria dos casos, no entanto, a uveíte pode ser crônica ou recorrente. Os autores descrevem três casos de síndrome nefrite tubulointersticial e uveíte, diagnosticados em idade pediátrica, e pretendem alertar para a necessidade de pesquisar sempre alterações renais nos doentes com uveíte.

## INTRODUCTION

Tubulointerstitial nephritis and uveitis (TINU) syndrome is a rare condition^1^^,^ 2 characterized by ocular and renal inflammation in the absence of other systemic diseases, recognized by diagnosis of exclusion.^1^^,^ 3 ^,^ 4 Diagnosing patients with TINU syndrome is no simple task, since the manifestations tied to the condition may not occur concurrently.^2^

The syndrome accounts for approximately 5% of the cases of tubulointerstitial nephritis^5^ and less than 2% of the cases of uveitis, and for a third of the cases of pediatric bilateral anterior uveitis.^1^^,^ 2

This paper describes three cases of pediatric TINU syndrome.

## CLINICAL CASES

### PATIENT 1

A 13-year-old female arrived at the Emergency Unit complaining of blurred vision and hyperemia, and pain in her right eye. She denied having fever, asthenia, anorexia, arthralgia, myalgia, ulcers, abdominal pain, lower back pain or urinary symptoms. The patient was diagnosed with bilateral nongranulomatous anterior uveitis without additional complications (Table 1). She had hypertension, raised levels of inflammatory markers (C-reactive protein: 2.6 mg/dL; erythrocyte sedimentation rate: 96 mm/first hour), a glomerular filtration rate (GFR) of 48 ml/min/1,73m^2^, hypokalemia, metabolic acidosis, leukocyturia, glucosuria, hematuria, non-nephrotic proteinuria, and raised urine β2-microglobulin levels. Hepatitis B and C, toxoplasmosis, brucellosis, Epstein-Barr virus (EBV) and cytomegalovirus (CMV) infection were ruled out. Her chest X-ray images were normal. Her angiotensin-converting-enzyme (ACE) levels were normal, and she was negative for antinuclear antibodies (ANA) and antineutrophil cytoplasmic antibodies (ANCA). Ultrasound imaging showed her kidneys were slightly enlarged. Kidney biopsy showed diffuse mononuclear cell interstitial infiltrates consistent with acute tubulointerstitial nephritis (Table 2). She was prescribed ocular dexamethasone and mydriatics, oral prednisolone (5 mg/m^2^/day), amlodipine, and potassium citrate; her blood pressure, serum creatinine, and tubular function were normalized, and she was on remission from uveitis within three months. Two months later she was started on methotrexate (10 mg/m^2^/week) on account of recurrent uveitis. She had two other episodes of recurrent uveitis without renal involvement, one and three years after being diagnosed; the dosages of methotrexate (12.5 mg/m^2^/week) and topical corticosteroids were adjusted. Five years after being diagnosed, the patient was asymptomatic and on methotrexate.

**Table 1:** Description of the three presented cases

		Case 1		Case 2		Case 3
Sex		Female		Female		Female
Age at diagnosis		13 years		12 years		12 years
Clinical presentation		Bilateral anterior nongranulomatous uveitis and hypertension		Bilateral anterior and intermediate uveitis		Asthenia, anorexia, polyuria, nocturia, and bilateral anterior and intermediate nongranulomatous uveitis with synechiae
Treatment		Ocular dexamethasone and mydriatics, oral prednisolone oral, methotrexate, amlodipine and potassium citrate		Ocular dexamethasone, prednisolone, and mydriatics, oral deflazacort and methotrexate		Ocular dexamethasone, prednisolone and mydriatics, oral prednisolone and methotrexate
Progress		Normal blood pressure, recovered renal function and remission from uveitis in three months		Normal renal function in six weeksRemission from uveitis in two months		Remission from uveitis in three weeks and improved renal function
Time on follow-up		Five years		Eighteen months		Five months
Follow-up		Three episodes of recurrent uveitis		No recurrence		One episode of recurrent uveitis

**Table 2:** Results of the complementary diagnostic tests performed in the described cases

	Case 1	Case 2	Case 3	Reference Values
**Analytical tests**				
Hemoglobin (g/dL)	12.9	10.8	11.1	12-15.3
ESR* (mm/first hour)	96	120	9	3-13
Leukocytes (/uL) - Neutrophils (/uL) - Eosinophils (/uL) - Basophils (/uL) - Lymphocytes (/uL) - Monocytes (/uL)	14890	9800	6380	4000 -11000
	11500	4900	3710	1900-7500
	400	800	240	0-500
	40	100	30	0-200
	2340	3200	1810	1000-4800
	540	800	590	100-1000
CRP* (mg/dL)	2.6	-	3.19	< 0.5
Urea (mg/dL)	41	36	61	16-49
Creatinine (mg/dL)	1.4	1.2	1.48	0.44-0.68
GFR* (ml/min/1.73m^2^)	48	47	47	> 90
Sodium (mmol/L)	138	137	140	135-145
Potassium (mmol/L)	3.0	4.0	4.1	3.5-5.1
Calcium (mg/dL)	9.4	9.5	9.4	8.6-10.2
Phosphorus (mg/dL)	3.1	4.2	4.3	2.5-6.0
AST* (U/L)	14	19	22	0-32
ALT* (U/L)	11	14	30	0-33
Total protein (g/dL)	8.7	8.7	7.1	6.4-8.2
Albumin (g/dL)	3.9	4.4	3.4	3.5-5.2
**Blood gas**				
pH	7.30	7.38	7.33	7.35-7.45
Bicarbonate (mmol/L)	21	22.5	22.6	22-26
**Urinalysis**				
pH	6.5	6.0	5.0	5.0-8.0
Density	1.010	1.013	1.018	1.015-1.025
Leukocytes (/uL)	125	25	25	Negative
Erythrocytes (/uL)	10	10	250	Negative
Glucose (mg/dL)	250	100	100	Normal
Protein (mg/dL)	100	75	75	Negative
24-hour urine protein (mg/m^2^)	366	240	-	Negative
Urinary excretion of β2-microglobulin (mg/L)	19.83	-	12.51	0.03-0.10
Renal histology	Diffuse interstitial mononuclear cell infiltrate. Immunofluorescence was negative.	Not performed	Lymphoplasmacytic interstitial infiltrate. Immunofluorescence was negative.	

* ESR: erythrocyte sedimentation rate; CRP: C-reactive protein; GFR: glomerular filtration rate; AST: aspartate transaminase; ALT: alanine transaminase.

### PATIENT 2

A 12-year-old female arrived at the Emergency Unit complaining she had been suffering from photophobia and ocular hyperemia for four weeks. She was diagnosed with bilateral anterior and intermediate uveitis (Table 1). Her blood pressure was normal; she had iron-deficiency anemia, an ESR of 120 mm/first hour, a GRF of 47 ml/min/1.73m^2^, leukocyturia, glucosuria, hematuria, and non-nephrotic proteinuria (Table 2). Infectious and autoimmune diseases were ruled out. Her chest X-ray images and kidney ultrasound examination did not show alteration. She was started on mydriatics, topical corticosteroids, and oral deflazacort. Her renal function recovered in six weeks and she was on remission from uveitis within two months of treatment; she stopped taking systemic corticosteroids and was started on methotrexate (10 mg/m^2^/week).

The patient was on methotrexate and asymptomatic 18 months after being diagnosed, and has not had renal dysfunction or recurrent uveitis.

### PATIENT 3

**Figure 1:**
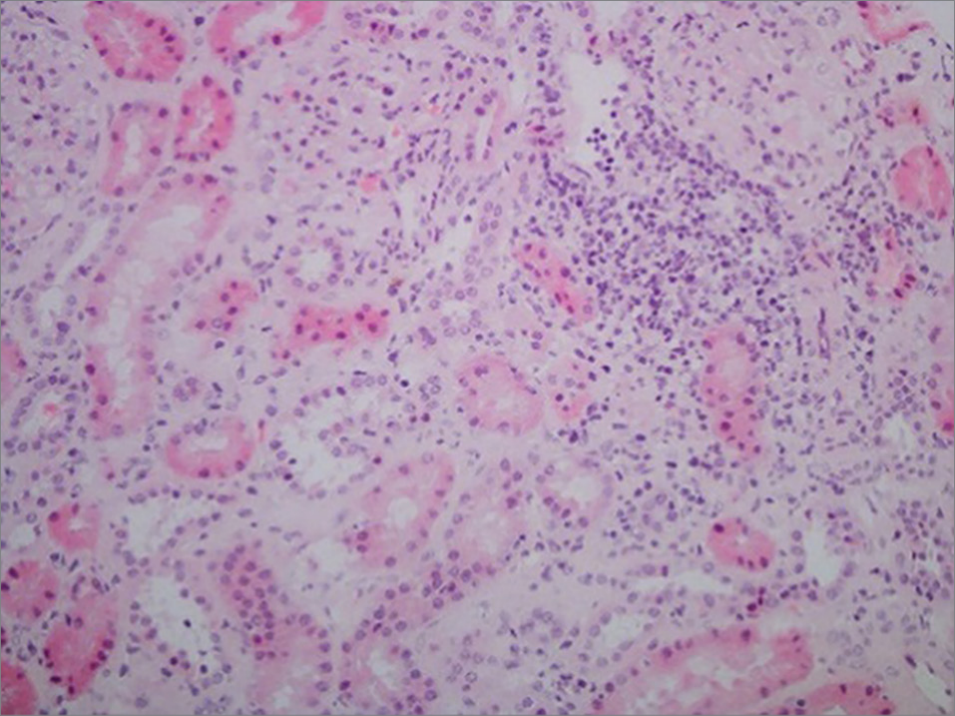
Renal histology for patient 1: renal interstices with lymphoplasmacytic inflammatory infiltrate.

A 12-year-old female arrived at the Emergency Unit with asthenia, anorexia, nocturia, polydipsia, normocytic normochromic anemia, and a GFR of 59 ml/min/1.73m^2^. She came in two months later with pain and hyperemia in her right eye, and was diagnosed with bilateral nongranulomatous anterior and intermediate uveitis with synechiae (Table 1). Her CRP was 3.19 mg/dL, the GFR was at 47 ml/min/1.73m^2^, and she presented with leukocyturia, glucosuria, hematuria, non-nephrotic proteinuria, and raised urine β2-microglobulin levels (Table 2). Infectious and autoimmune diseases were ruled out. Her chest X-ray images were normal. Renal histology showed lymphoplasmacytic interstitial infiltrates consistent with acute tubulointerstitial nephritis (Figure 1). She was treated with ocular corticosteroids and mydriatics, oral prednisolone (12.5 mg/m^2^/day), and methotrexate up to 15 mg/m^2^/week. She was on remission from uveitis three weeks after being diagnosed and her renal function improved. Two months later she had recurrent uveitis and was started again on topical corticosteroids. She entered remission and was on methotrexate.

## DISCUSSION

This report described three cases of TINU syndrome, a rare condition with few published cases.^1^^,^ 3 ^,^ 6

Incidence is greater in females (3:1).^7^ Younger individuals with a median age of 15 years at the time of diagnosis are preferentially affected,^7^^,^ 8 as seen in our series.

The pathogenesis of the disease is still unclear.^4^ Associations between drugs and infection have been established in a few cases, including non-steroid anti-inflammatory drugs and antibiotics with infections by *Mycobacterium tuberculosis, Toxoplasma gondii*, and the Epstein-Barr and Varicella-Zoster viruses.^3^^,^ 8 The presence of autoantibodies against modified C-reactive protein in the kidneys was recently described.^9^

The criteria used to investigate TINU syndrome allow the establishment of definitive diagnosis when anterior uveitis is present, with or without the involvement of the intermediate or posterior segments, with onset two months before or 12 months after tubulointerstitial nephritis. Nephritis may be diagnosed based on clinical criteria, including factors such as decreased GFR, alterations in urinalysis (raised urine β2-microglobulin levels, hematuria, non-nephrotic proteinuria, glucosuria, pyuria, urine eosinophils and leukocyturia), and systemic alterations (fever, asthenia, weight loss, exanthema, abdominal and flank pain, arthralgia, myalgia and laboratorial findings such as anemia, eosinophilia, increased ESR and changes in liver function).^1^ Albeit not mandatory, renal biopsy may be performed to confirm the diagnosis.^1^^,^ 4

Uveitis is generally of the bilateral nongranulomatous and anterior type,^2^^,^ 4 as seen in the cases described herein. Vitreous involvement may also occur,^2^^,^ 4 as seen in patients 2 and 3.

Tubulointerstitial nephritis may be asymptomatic or associated with systemic symptoms, arthralgia, myalgia, abdominal and back pain, and nocturia.^1^^,^ 2 ^,^ 3 It precedes uveitis in 65% of the cases.^4^^,^ 7 In patients 1 and 2, renal function was assessed only after the patients had been diagnosed with uveitis; then the diagnosis of tubulointerstitial nephritis was established. 

Urinary excretion of β2-microglobulin is increased in 87% of the cases due tubular reabsorption defects. The test used to measure urine levels of β2-microglobulin is not invasive and offers diagnostic sensitivity and specificity of 88% and 70%, respectively. The predictive value of the test is significantly enhanced by a combination of decreased GFR and increased urine β2-microglobulin levels.^1^

Biopsy allows the confirmation of nephritis,^1^ as seen in the cases of patients 1 and 3. However, the diagnosis of TINU syndrome may be established without the aid of biopsy if the clinical criteria for tubulointerstitial nephritis are present, as in the case of patient 2.

Differential diagnosis includes other conditions that manifest with tubulointerstitial nephritis and uveitis, namely sarcoidosis, systemic lupus erythematosus, granulomatosis with polyangiitis, Behçet's disease, syphilis, tuberculosis, and brucellosis.^1^^,^ 3 ^,^ 4

Definitive diagnosis of TINU syndrome was reached in the three patients after other systemic conditions were ruled out.

In the absence of complications in the posterior segment, the treatment of uveitis initially includes mydriatics and topical corticosteroids; therapy with oral corticosteroids and/or other immunosuppressants may be needed in cases of refractory or recurrent disease.^2^^,^ 4 Immunosuppressant therapy, specifically with methotrexate, mycophenolate mofetil, azathioprine or cyclosporine is indicated in cases of resistance to treatment with corticosteroids, recurrent uveitis or adverse effects associated from the use of corticosteroids.^4^^,^ 10

Uveitis may be chronic or recurrent in up to 50% of the cases,^1^^,^ 2 ^,^ 4 as seen in patients 1 and 3. Recurrence has been reported as many as ten years after initial diagnosis.^11^ Affected patients must be followed for possible ophthalmic involvement. Ocular complications such as synechiae, optic disc edema, cataract, and glaucoma have been described in 21% of the cases.^7^

Renal disease is usually self-limited and resolves spontaneously or after treatment with corticosteroids.^2^ However, 10% progress to chronic kidney disease and may require renal replacement therapy.^7^ Progression to chronic kidney disease has been associated with delays in the start of therapy with systemic corticosteroids.^12^

Therefore, the authors believe that all patients with uveitis should be suspected and screened for renal involvement, so that they are accurately diagnosed and treated in a timely manner to prevent the progression of kidney disease and the onset of ocular complications.
